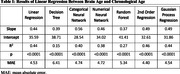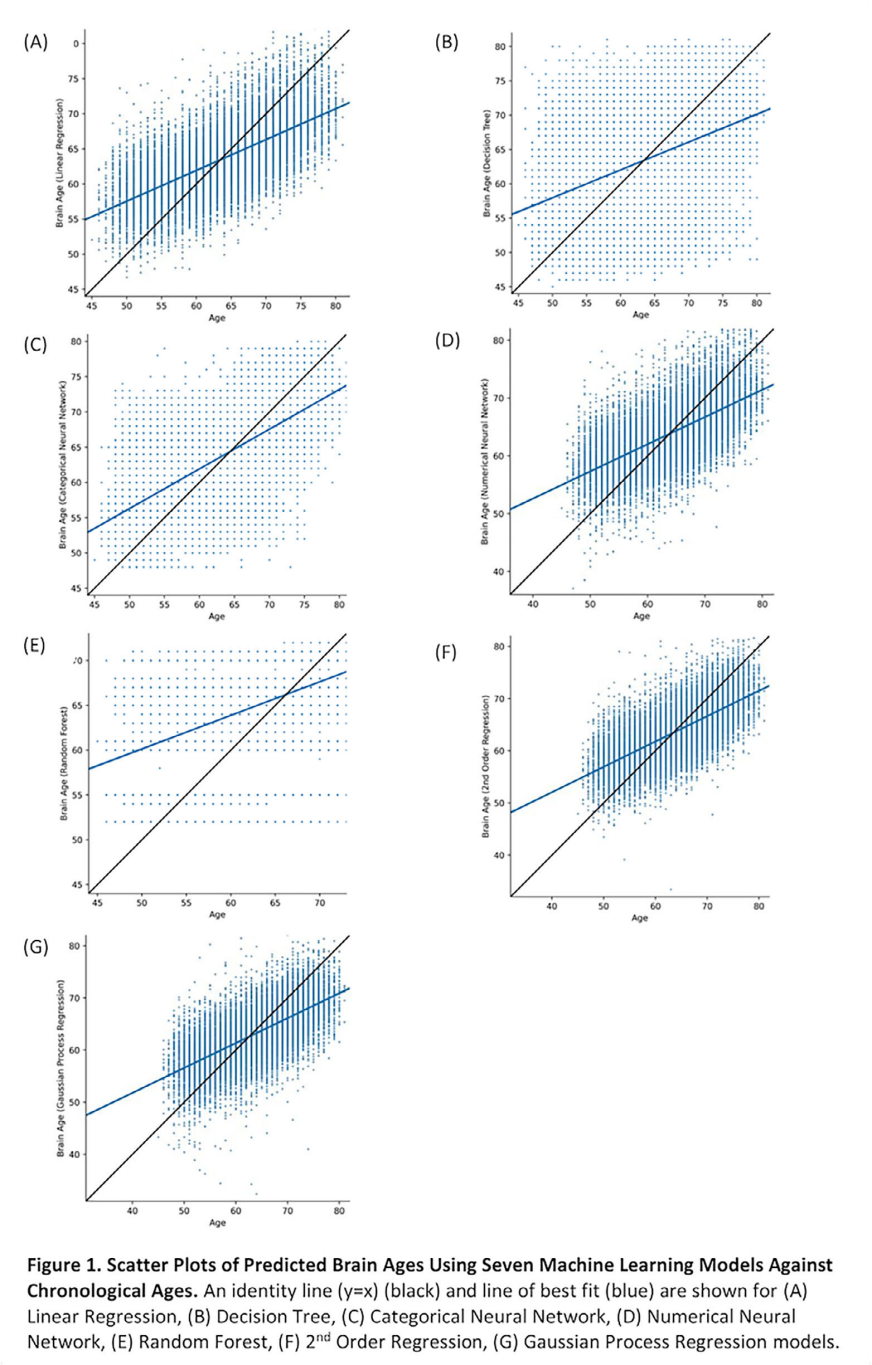# Development and Preliminary Feasibility of a Translatable Volumetric Brain Age Metric

**DOI:** 10.1002/alz.093359

**Published:** 2025-01-09

**Authors:** Gavin T Kress, Juan Eugenio Iglesias, James H. Cole, Emily S. Popa, Prabha Siddarth, Jennifer E. Bramen

**Affiliations:** ^1^ The Icahn School of Medicine at Mount Sinai, New York, NY USA; ^2^ Pacific Brain Health Center, Pacific Neuroscience Institute and Foundation, Santa Monica, CA USA; ^3^ Martinos Center for Biomedical Imaging, Massachusetts General Hospital and Harvard Medical School, Boston, MA USA; ^4^ Centre for Medical Image Computing, University College London, London UK; ^5^ Computer Science and Artificial Intelligence Laboratory, Massachusetts Institute of Technology, Cambridge, MA USA; ^6^ UCL, London UK; ^7^ David Geffen School of Medicine at University of California Los Angeles, Los Angeles, CA USA; ^8^ Saint John's Cancer Institute at Providence Saint John's Health Center, Santa Monica, CA USA

## Abstract

**Background:**

Brain aging (BA) involves the gradual deterioration of brain systems and is associated with chronological age (CA). Measures of BA have been validated and adopted in aging and neurological disease research (Biondo, 2022; Eickhoff, 2021) and could be a useful clinical tool. BA predicts CA in healthy adults (Cole, 2017) and accelerated BA precede Alzheimer’s disease (AD) symptoms (Elliott, 2021). Current BA estimations rely on the acquisition of high‐quality neuroimaging data and complex, computationally expensive analysis workflows, creating obstacles for clinical translation. This study aims to develop an easily implementable workflow for BA where inputs are clinically relevant features derived from heterogeneously acquired magnetic resonance imaging (MRI) using the SynthSeg tool.

**Method:**

Thirty‐two regional volumes from T1‐weighted MRIs from 56,505 participants (mean age=63.6; 44‐82; SD=7.52) were segmented using the SynthSeg tool. This tool analyzes clinically acquired, heterogenous inputs and requires minimal processing power, memory, and time. Only z‐transformed SynthSeg‐derived measures were included to allow investigations into confounds. Performance evaluation included three filter‐based feature selection approaches (Fischer’s Score, Information Gain, and Correlation Coefficient) combined with seven machine learning (ML) algorithms including linear regression (LR), 2^nd^‐order polynomial regression (2PR), Gaussian process regression (GPR), Decision Tree (DT), Random Forest (RF), a Convolutional Neural Network (CNN) treating age as a continuous variable, and as a categorical measure. Ability to predict CA was assessed through 10‐fold cross validation, each with an independent 90‐10 training‐testing split, and regression analysis between predicted and CA using an alpha=0.001.

**Result:**

Feature selection demonstrated that each structural feature contained unique information for predicting CA. All seven ML algorithms significantly predicted CA (Table 1). 2PR performed the best overall with LR and GPR following closely behind (Figure 1). Both the categorical and numerical CNN performed slightly worse, but still significantly better than the RF and DT

**Conclusion:**

The association between CA and volumetric‐BA estimates derived from SynthSeg segmented data supports the potential validity of this approach. However, its simplicity may increase translatability. Future directions of this work are to improve performance. The long‐term goal of this work is to develop and validate local volumetric‐BA (as opposed to global volumetric‐BA) metrics.